# Improving spine‐based age estimation in centrarchid fishes using otolith‐derived training sets

**DOI:** 10.1111/jfb.70452

**Published:** 2026-04-22

**Authors:** Matthew P. Woo, Stuart E. Jones, Brian S. Ankrapp, Avi Neumann, Julián Torres‐Dowdall

**Affiliations:** ^1^ Department of Biological Sciences University of Notre Dame Notre Dame Indiana USA; ^2^ Robert B. Annis Water Resources Institute, Grand Valley State University Muskegon Michigan USA; ^3^ Department of Ecology and Evolutionary Biology University of Michigan Ann Arbor Michigan USA; ^4^ College of Food, Agricultural and Natural Resource Sciences University of Minnesota St. Paul Minnesota USA

**Keywords:** age estimation, bluegill, centrarchids, dorsal spine, largemouth bass, otolith

## Abstract

Nonlethal age determination is a priority in fish biology contexts where sacrifice is restricted, but estimates derived from nonlethally attainable structures tend to suffer from poor precision and accuracy relative to necessarily lethal, otolith‐derived estimates. Dorsal spines, while less accurate than otoliths, have shown higher precision compared to other alternative structures, making them a useful candidate for testing reader‐based approaches to improved age estimation. Here, we test whether small, lake‐specific training sets derived from paired otolith‐spine images, derived from incidental mortalities can provide useful calibration to spine‐based age estimates in four centrarchid populations from the northern Midwest, including two largemouth bass (*Micropterus nigricans*) and two bluegill (*Lepomis macrochirus*) populations. Five moderately experienced readers estimated ages from spine images before and after calibration with paired otolith‐spine images. Calibration consistently resulted in increased estimated ages, particularly for older fish, and reduced variability among readers in both individual age estimates and predicted length‐at‐age trajectories. Specifically, we find that these structured training sets are useful in both reducing traditional underestimation biases and reducing reader‐based variance effects at both the level of individual estimates and in growth modelling contexts. Therefore, they may offer a practical means of leveraging available incidental mortalities in the improvement of otherwise nonlethal aging programs. Accordingly, we suggest that where available, readers first be familiarized with lake‐specific paired otolith‐spine images before independently estimating spine ages.

## 1 INTRODUCTION

The accurate determination of age represents a crucial component of fish and fisheries research. Because age data are used to inform growth, mortality, year‐class strength and many other parameters important to fisheries science, error in age determination propagates to a variety of downstream demographic and ecological inferences (Robillard & Ellen Marsden, 1996). Fish age data are most often generated by analysing calcified structures, which can display growth intervals associated with annual patterns (Maceina et al., 2007). However, evidence suggests that among species, different structures can vary substantially in their capacities to yield accurate annuli counts. For instance, various catostomid and cyprinid fishes have been shown to be aged most accurately using fin rays (Quist et al., 2007), while percid and centrarchid fishes have been shown to be most accurately aged using sagittal otoliths (Buckmeier & Howells, 2003; Isermann et al., 2003; Sotola et al., 2014). These effects are further complicated by differences in reader experience and training, of which effects have not been well characterized.

Centrarchid fishes, originally endemic to eastern North America, include popular species such as black basses and bluegill, and collectively contribute to globally significant recreational fisheries and aquaculture programs (Cooke & Philipp, 2009). For instance, the management of black basses (*Micropterus* spp.) in North America can be traced back over 220 years to 1802, beginning with the movement of large individuals between lakes to enhance angling opportunities (Long et al., 2015). Because centrarchid fishes have been shown to exhibit remarkably variable growth rates (Sotola et al., 2014), accurate in situ age data are especially important in deriving demographic and ecological assessments.

A review by Maceina et al. (2007) found that among 45 American and Canadian management agencies, ~60% used scales to age black basses, ~35% used otoliths and ~5% used dorsal spines. Similarly, ~55% used scales to age sunfishes, 40% used otoliths and fewer than ~3% used dorsal spines or fin rays (Maceina et al., 2007). While at least five different aging structures have been used to age centrarchid fishes (Sotola et al., 2014), available evidence continues to suggest that relative to other structures, otoliths are more robust to the loss of annuli, and thus represent the most precise and accurate means of age determination available (Buckmeier & Howells, 2003; Kowalewski et al., 2012; Kruse et al., 1993; Long & Fisher, 2001; Perrion et al., 2024). Although mark‐recapture methods have been suggested to offer further improvements compared to otolith‐based estimates (Doering‐Arjes et al., 2008; Osborne et al., 2021), they are often infeasible or intractable, causing otoliths to remain a widely accepted method for centrarchid age determination.

Because otoliths require fish sacrifice, and since centrarchid fishes are sometimes managed in systems where fish sacrifice is unacceptable (Long & Fisher, 2001), robust nonlethal age determination remains a priority. While most nonlethal structures are thought to systematically underestimate ages for fish beyond 5–8 years of age, several studies report substantial precision and accuracy differences. In a Northern Indiana largemouth bass (*Micropterus nigricans*) study, Morehouse et al. (2013) found dorsal spines to yield reductions in both bias and variability compared to fin rays and scales, suggesting some promise for the use of dorsal spines. Sotola et al. (2014) also found dorsal spines to yield lower between‐reader estimate variation compared to scales among black basses, as did Isermann et al. (2010) among black crappie (*Pomoxis nigromaculatus*). However, authors have argued that the observation of poor agreement between spine‐derived and otolith‐derived estimates suggests that spines, regardless of precision, may not be useful due to their poor accuracy and persistent underestimation of older fish (Isermann et al. 2010, Klein et al., 2017). To our knowledge, no studies have explored approaches for reducing the bias of these estimates.

Despite these constraints, the persistent demand for nonlethal age determination raises the question of whether the systematic biases associated with nonlethal structures can be understood, predicted and corrected in a manner that meaningfully reduces discrepancies relative to estimates derived using more robust methods. Spines are thought to systematically generate lower age estimates compared to otoliths because of increased susceptibility to the resorption and occlusion of early annuli, of which the latter is often attributed to expansion of the vascular core (Erickson, 1983; Klein et al., 2017). In both cases, early increments are faint or obscured by subsequent growth, resulting in failures to be detected by readers. While the factors that govern the onset of annulus resorption or occlusion are not well understood, the observation that spine biases tend to strengthen as fish approach asymptotic lengths (Erickson, 1983) suggests that the relationship between age and the extent of resorption/occlusion may be at least partly correlated with somatic growth dynamics. Because these biases may be systematic, they may be predictable, and potentially correctable if small numbers of otoliths collected from the same lakes are used to train spine‐based readings.

Here, we tested whether the use of small numbers of otolith samples (11–16), obtained from incidental sampling fish mortality and used in paired otolith‐spine image sets, can serve as within‐lake tools for the calibration of spine‐generated centrarchid age estimates. Specifically, we evaluate whether these training sets, designed to familiarize readers with local annulus patterns, can improve precision and accuracy relative to uncalibrated spine estimates, and offer a potential means of leveraging available incidental mortalities in enhancing otherwise nonlethal aging programs.

## 2 METHODS

### 2.1 Fish sampling

Dorsal spines were collected during mark‐recapture surveys of two largemouth bass (*Micropterus nigricans*, Cuvier, 1828) populations and two bluegill (*Lepomis macrochirus*, Rafinesque, 1810) populations in the Upper Midwest United States, near the Michigan–Wisconsin border. Bluegill were sampled from Deadwood Lake (Vilas County, WI, USA) and Hummingbird Bog (Gogebic County, MI, USA), while largemouth bass were sampled from Jones Lake and Crampton Lake, both in Vilas County, WI. Across all populations, dorsal spines were taken from unique individuals ranging in size from approximately 40 mm to the largest specimens available (227–251 mm for bluegill and 451–513 mm for largemouth bass), although spines from small largemouth bass (total length <50 mm) proved difficult to process due to their susceptibility to breakage.

Dorsal spines were excised from fish using scissors, as close to the base as possible (Watkins et al., 2015). When available, the second‐most anterior dorsal spine was collected, but if damaged, or if visible pathologies were present, the third‐most anterior spine was substituted. In Jones Lake, 83 dorsal spines were collected from largemouth bass 50–513 mm in length, while otoliths were obtained from 11 incidental mortalities ranging from 229 to 305 mm in length. In Crampton Lake, 80 dorsal spines were collected from largemouth bass 61–451 mm in length, while otoliths were obtained from 12 incidental mortalities ranging from 55 to 398 mm in length. In Hummingbird Bog, 58 dorsal spines were collected from bluegill 43–220 mm in length, while otoliths were obtained from 16 incidental mortalities ranging from 114 to 196 mm in length. In Deadwood Lake, 66 dorsal spines were collected from bluegill 47–251 mm in length, while otoliths were obtained from 14 incidental mortalities ranging from 49 to 227 mm in length. The total number of mortalities in this study (53) remained under 0.6% of all captures in an unrelated study with 8000+ capture events.

### 2.2 Structure processing

Following collection, dorsal spines were placed in standard coin envelopes to dry completely for a minimum of 2 weeks before further processing. Once dried, spines were embedded in a 2 mm thick malleable plastic sheet and sectioned a single time, as close to the base as possible (<1 mm), manually using a jewellery saw. Embedded sections were then sanded manually using standard 1000‐grit sandpaper lubricated with 70% ethanol. Sanding was repeated iteratively until a smooth surface could be imaged. Embedded sections were finally blotted with mineral oil and imaged under a standard dissecting microscope with transmitted light (Schram, 1989).

Otoliths, once extracted, were similarly placed in standard coin envelopes to dry completely for over 2 weeks before further processing. Once dried, otoliths were manually cracked widthwise about the core, after which cracked faces were roasted near an open flame (Vagenas & Karachle, 2021). Roasted halves were then embedded in modelling clay (Barber & McFarlane, 1987) and cracked faces were blotted with mineral oil and imaged under a standard dissecting microscope with incident light.

### 2.3 Age estimation

**FIGURE 1:**
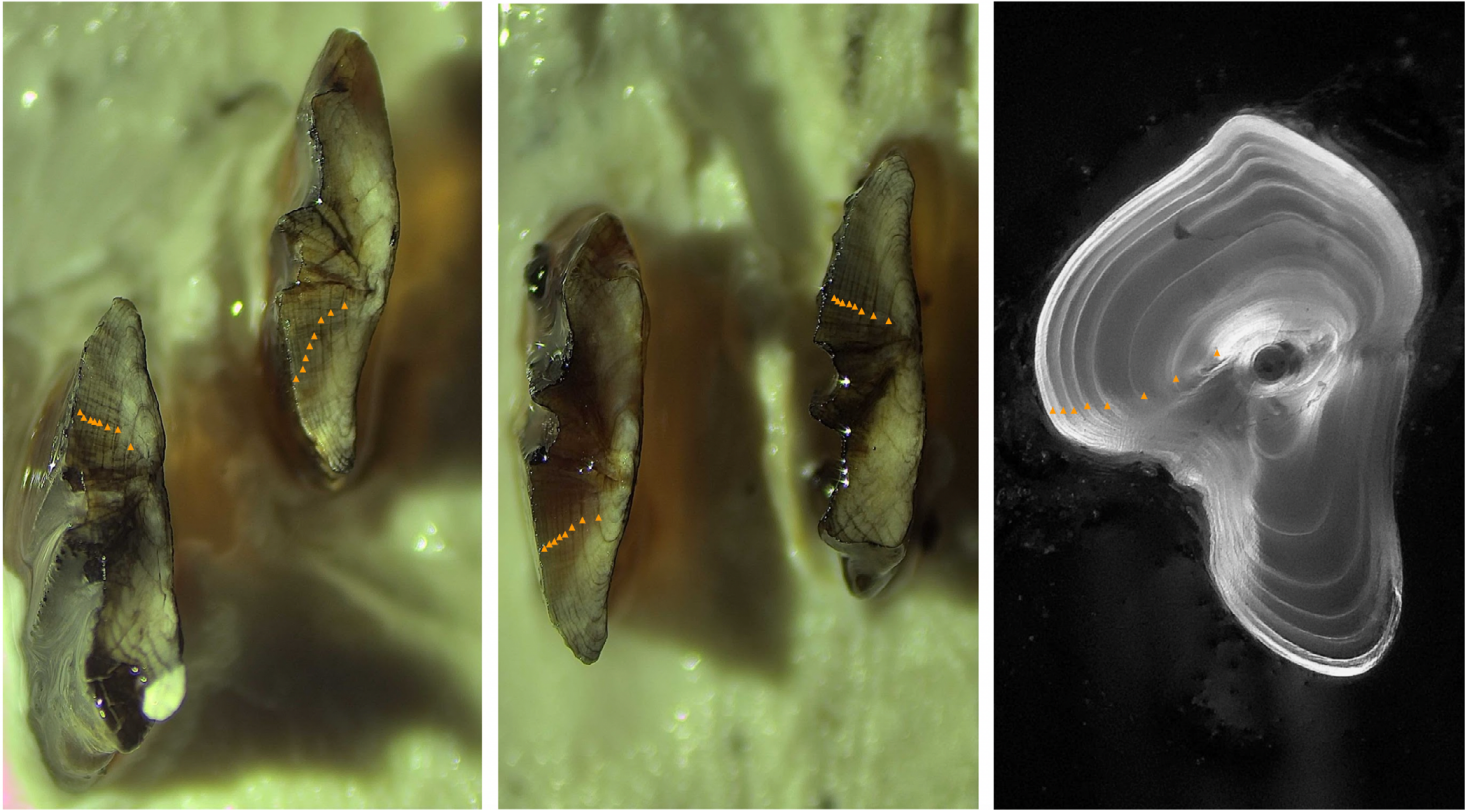
An example of a single paired otolith‐spine image (otoliths left, spine right) set used in calibration, derived from a 398 mm largemouth bass mortality in Crampton Lake, marked and estimated at age 8 years+ by reader R3. As with all paired image sets, readers were forced to derive a consensus estimate, and based on otolith annuli patterns, to mark an identical number of annuli on all spine images provided. All five readers agreed on the same estimate for this image set.

To determine whether structured otolith‐derived training sets could improve the calibration of spine‐derived age estimates, we designed a paired experiment where spine images were individually evaluated before and after a round of reader calibration. Paired otolith‐spine image groups (Figure 1), used in the experiment's calibration phase, were compiled separately from independent spine images. Five amateur readers, with modest training (<2 months of experience), were used for the generation of pre‐ and post‐calibration age estimates. Readers were trained together, using the same basic instructions regarding the identification of annuli, the detection of faint annuli and the avoidance of phantom and double annuli.

For each lake, independent spine images were shown to readers in a randomized order and readers were instructed to mark annuli on the images, recording an age estimate in ‘years+’, not accounting for the summer growth interval active at the time of collection, and a confidence rating, scaled from 1 to 4. A rating of level 1 corresponded to ‘this estimate is almost a guess’. A rating of level 2 corresponded to ‘I feel moderate about my estimate, and understand how someone else might generate a reading different by a few years in either direction’. A rating of level 3 corresponded to ‘I am confident in my estimate, but could understand how someone else might generate a reading different by one year in either direction’. Finally, a rating of level 4 corresponded to ‘“I am sure of my estimate and would disagree strongly with any other readings’.

Next, readers were shown paired otolith‐spine image sets from individual incidental mortalities and instructed to mark annuli on the images such that each reader arrived at a single consensus age estimate for the entire set, and such that the number of annuli marked on the spine images was forced to match the number marked on the otolith images (Figure 1). Finally, readers were provided the same independent spine images in a randomized order and were instructed to mark annuli on the images, similarly recording an age estimate and a confidence rating, scaled from 1 to 4. The above process was repeated among readers for each lake, and the order in which lakes were assigned to readers was randomized. This ensured that systematic learning effects or sequential biases did not influence the observed calibration effects and that improvements reflected lake‐specific otolith‐spine relationships derived from same‐lake otoliths rather than transferable patterns learned from other populations.

### 2.4 Statistical analysis

To measure the effects of calibration on age estimates, while accounting for repeated measurements and hierarchical data structure, we fit a linear mixed‐effects model of the following form using the R package *lme4* (Bates et al., 2015):

$$\mathrm{Ag}e_{\text{ijkl}}=\begin{array}{l} \beta_{0}+\beta_{1}RD_{i}+\beta_{2}MA_{j}+\beta_{3}L_{k}+\beta_{4}RD_{i}*\mathrm{Lak}e_{k}+\beta_{5}RD_{i}*MA_{j}\\ +\;u_{l}^{\text{(Reader)}}{+u}_{m}^{\text{(Fish)}}+e_{\text{ijkl}} \end{array}$$

where $\mathrm{Ag}e_{\text{ijkl}}$ is the observed age of fish of mean estimated age (MA) *j* in lake (L) *k* as read by reader *l* under round (RD) *i* (uncalibrated vs. calibrated). Fixed effects (*ꞵ*) included round, mean estimated age, lake and their interactions, representing population‐level effects. Random effects (*μ*) accounted for repeated measurements of individual fish and variability among readers. Model assumptions (normality and homoscedasticity of residuals) were assessed visually with residual vs. fitted plots. No substantial violations or influential outliers were observed. Estimated marginal means (EMMs) were computed using the R package *emmeans* (Lenth, 2024) to evaluate pairwise differences between calibrated and uncalibrated age estimates in each lake, with Tukey adjustments for multiple comparisons.

To characterize the effects of calibration on estimate consistency among readers, we computed per‐fish agreement metrics across all readers for each round and lake. Specifically, we measured mean coefficient of variation (CV) (Campana et al., 1995; Chang, 1982), complete agreement (cases in which all readers assigned the same age) and partial agreement (cases in which all reader ages existed within ±1 year, as defined in multiple studies, including Sotola et al. (2014), Lindelien et al. (2024) and Morehouse et al. (2013)). Accordingly, cases of partial agreement also include all those of complete agreement.

Furthermore, to evaluate how calibration influenced readers' perceptions of their own performance, and whether confidence aligned with actual precision, we conducted two analyses. First, we compared readers' self‐reported confidence scores before and after calibration, assessing whether paired confidence values on repeated images deviated from a null expectation of no change, using paired Wilcoxon signed‐rank tests. Second, to determine whether confidence provided real information about estimation precision, we quantified the association between mean confidence across all readers and CV in age estimates of individual fish using Spearman's rank correlation.

Finally, to consider the consequences of using calibrated and uncalibrated age estimates in formal growth analyses, we generated predicted length‐at‐age (LAA) metrics based on von Bertalanffy growth functions per reader, lake and round. Predicted length values (per‐age) were evaluated across a sequence of ages spanning from 0 to the highest age estimated by any single reader, and mean pairwise Euclidean distances among readers' predicted lengths were computed at each age. These distances provide an integrative measure of reader agreement in the context of growth models, accounting for differences in all three VBGF parameters (*L*_∞_, *K* and *t*_0_). Paired Wilcoxon signed‐rank tests were used to evaluate whether calibration systematically reduced divergence in predicted LAA among readers in each lake.

## 3 RESULTS

### 3.1 Age estimate population

Among the four populations considered and across all spines and incidental otoliths, bluegill age estimate ranges spanned from 0 to 7 in Hummingbird Bog and 0–18 in Deadwood Lake, and largemouth bass spanned from 0 to 20 in Crampton Lake and 0–21 in Jones Lake. Hummingbird Bog bluegill were characterized by fast, sustained growth, but where elevated rates of mortality precluded survival to near‐asymptotic sizes. Conversely, Deadwood Lake bluegill were generally characterized by slower growth, but a greater abundance of larger, older individuals. Largemouth bass through asymptotic sizes were available from both Jones Lake and Crampton Lake, which for their part exhibited similar asymptotic sizes, but Jones Lake exhibited a substantially lower growth rate than Crampton Lake (Figure 1).

### 3.2 Effects of calibration on age estimates

**FIGURE 2:**
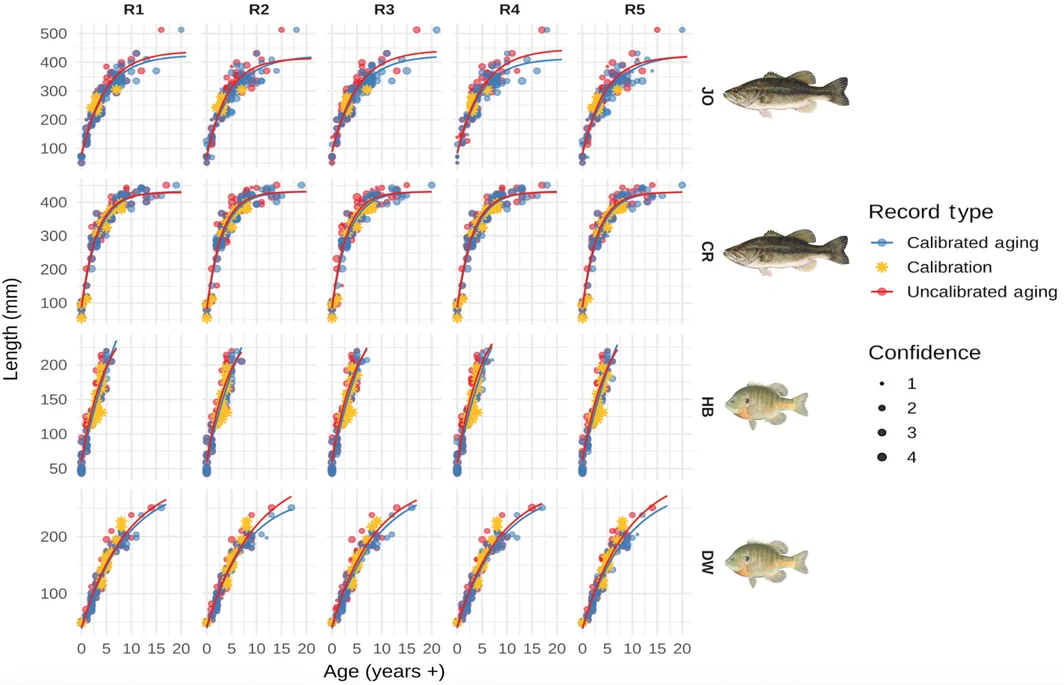
Age‐length data estimated from dorsal spines by individual readers (R1–R5) in both uncalibrated (red) and calibrated (blue) rounds, alongside associated von Bertalanffy growth functions. Yellow stars represent age estimates derived from paired otolith‐spine training sets. Columns represent readers and rows represent lakes. JO, Jones Lake, largemouth bass; CR, Crampton Lake, largemouth bass; HB, Hummingbird Bog, bluegill; DW, Deadwood Lake, bluegill. Largemouth bass and bluegill illustrations used as figure labels were adapted from solrman (CC0 1.0), via Wikimedia Commons, and from the Freshwater and Marine Image Bank, University of Washington (public domain), respectively.

**FIGURE 3:**
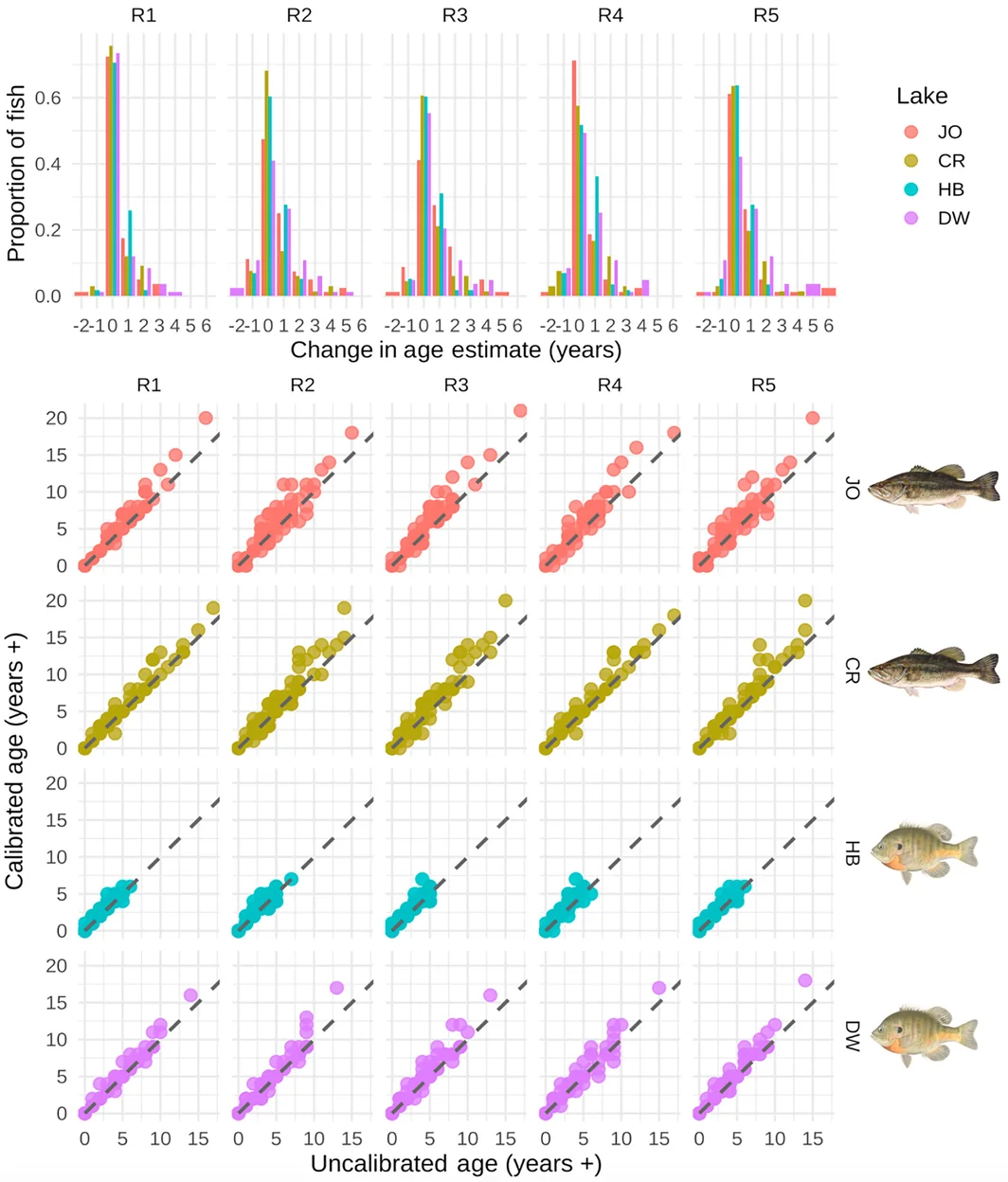
Changes in age estimates of particular fish among lakes and readers by frequency of magnitude and by initial uncalibrated age estimate. Rows represent lakes. JO, Jones Lake, largemouth bass; CR, Crampton Lake, largemouth bass; HB, Hummingbird Bog, bluegill; DW, Deadwood Lake, bluegill. In general, readers (R1–R5) exhibited some degree of variability in fidelity to their original estimates, but where changes existed, estimate increases were more frequent than decreases, and tended to exist more often for older individuals. Largemouth bass and bluegill illustrations used as figure labels were adapted from solrman (CC0 1.0), via Wikimedia Commons, and from the Freshwater and Marine Image Bank, University of Washington (public domain), respectively.

Calibration produced a consistent, significant effect on age estimates in all lakes (Figures 2 and 3). In line with expectations, calibrated ages were older than uncalibrated ages (*F*_1,2856_ = 39.4, *p* < 0.0001), with the magnitude of effects varying among lakes (Round × Lake interaction: *F*_3,2856_ = 8.19, *p* < 0.0001) and increasing with the mean estimated age of an individual fish (Round × Mean Estimated Age interaction: *F*_1,2856_ = 656.3, *p* < 0.0001), providing evidence of stronger calibration effects for older fish. EMMs indicated that calibration increased predicted ages by 0.374–0.612 years depending on the lake (Table 1). Random effects for individual fish were negligible, indicating that individual fish contributed little unexplained variability beyond mean estimated age. Reader effects were minimal (*σ* ^2^[reader] = 7.98*e* ^−5^). Most of the remaining variation was captured by the residual (*σ* ^2^[residual] = 0.263), reflecting among‐reader disagreement on individual fish.

**TABLE 1:** Differences in estimated marginal means (EMMs) of spine‐based age estimates between uncalibrated and otolith‐calibrated readings in four centrarchid populations. Significance codes are found in the footnote.

Population	Estimate change	SE	*df*	*t* ratio	*p*
Jones Lake (largemouth bass)	0.545	0.0357	2779	−15.296	<0.0001****
Crampton Lake (largemouth bass)	0.400	0.0366	2779	−10.923	<0.0001****
Hummingbird Bog (bluegill)	0.618	0.0441	2779	−14.019	<0.0001****
Deadwood Lake (bluegill)	0.374	0.0399	2779	−9.369	<0.0001****

*Note*: Degrees of freedom are estimated using the Kenward–Roger approximation.

Abbreviation: SE, standard error.

* Significant at 0.05 level.

** Significant at 0.01 level.

*** Significant at 0.001 level.

**** Significant at 0.0001 level.

### 3.3 Reader agreement

Complete reader agreement among individual uncalibrated estimates ranged from 42.2% (Jones Lake, largemouth bass) to 51.5% (Deadwood Lake, bluegill), while partial agreement (cases in which all reader ages existed within ± 1 year) among uncalibrated estimates ranged from 87.9% (Deadwood Lake, bluegill) to 94.8% (Hummingbird Bog, bluegill). Uncalibrated mean CV ranged from 6.5 (Deadwood Lake, bluegill) to 20.9 (Hummingbird Bog, bluegill). All three aforementioned metrics improved consistently following calibration in all lakes except for Jones Lake. In Jones Lake, complete agreement increased following calibration, but mean CV and partial agreement appeared to decrease in quality (Table 2). Among calibrated estimates, complete reader agreement ranged from 48.2% (Jones Lake, largemouth bass) to 75.9% (Hummingbird Bog, bluegill), while partial agreement ranged from 87.9% (Jones Lake, largemouth bass) to 100.0% (Hummingbird Bog, bluegill). Mean CV among calibrated samples ranged from 3.7 (Deadwood Lake, bluegill) to 10.9 (Jones Lake, largemouth bass).

**TABLE 2:** Reader agreement metrics including mean coefficient of variation (CV), complete agreement (cases in which all readers assigned the same age) and partial agreement (cases in which all reader ages existed within ±1 year) among uncalibrated and calibrated age estimates in four centrarchid populations.

Population × round	Mean CV		Complete agreement (%)		Partial agreement (%)		*n*
	Uncalibrated	Calibrated	Uncalibrated	Calibrated	Uncalibrated	Calibrated	
Jones Lake (largemouth bass)	9.1	10.9	42.2	48.2	89.2	88.0	83
Crampton Lake (largemouth bass)	13.0	7.9	45.0	53.8	88.8	96.3	80
Hummingbird Bog (bluegill)	20.9	8.9	48.3	75.9	94.8	100	58
Deadwood Lake (bluegill)	6.5	3.7	51.5	66.7	87.9	97.0	66

*Note*: Metrics in parentheses are associated with calibrated estimates. Agreement metrics exhibit consistent qualitative improvement in all populations with the exception of Jones Lake.

### 3.4 Reader confidence

We found inconsistent evidence of improvement in self‐reported reader confidence following calibration. Wilcoxon signed‐rank tests (all *n*[paired estimates] = 287) indicated that confidence shifts varied among readers: R1 (*V* = 136, *p* < 0.0001) and R3 (*V* = 3685.5, *p* = 0.0113) showed significant, but opposite, changes, whereas R2, R4 and R5 exhibited no significant differences (all *p* > 0.05). We furthermore detected a significant but weak negative relationship between mean confidence ratings and CV in assigned ages (*ρ* = −0.116, *p* = 0.0077), suggesting a modest association between confidence and precision.

### 3.5 Consequences for growth modelling

In the context of growth modelling, mean predicted LAA values derived from calibrated estimates were lower for almost all ages, in all lakes, than those derived from uncalibrated estimates. Cases where predicted LAA was higher when derived from calibrated estimates occurred seldom and only near boundary length values near age 0, or near the maximum observed length values in a lake's given age‐length dataset (Table 3).

**TABLE 3:** Mean predicted length‐at‐age (LAA), in mm, for four centrarchid populations generated from calibrated and uncalibrated dorsal spine estimates.

	Largemouth bass				Bluegill			
	Jones Lake		Crampton Lake		Hummingbird bog		Deadwood Lake	
Age	Pre‐	Post‐	Pre‐	Post‐	Pre‐	Post‐	Pre‐	Post‐
1	148	148	182	175*	99	91*	69	66*
3	246	236*	302	291*	158	146*	116	112*
5	311	296*	364	355*	197	191*	154	148*
7	353	337*	396	390*	224	228	184	177*
9	380	365*	413	409*			209	200*
11	398	383*	421	419*			228	218*
13	410	396*	426	425*			244	232*
15	418	405*	428	428			256	244*
17	423	411*	429	430			266	253*
19	426	415*	430	431				
21	428	417*						

*Note*: Calibrated ages are bolded, italicized and asterisked across the age range in which calibration proved able to reduce estimated LAA, and therefore potentially useful in reducing the systematic underestimation of ages traditionally associated with spines.

**FIGURE 4:**
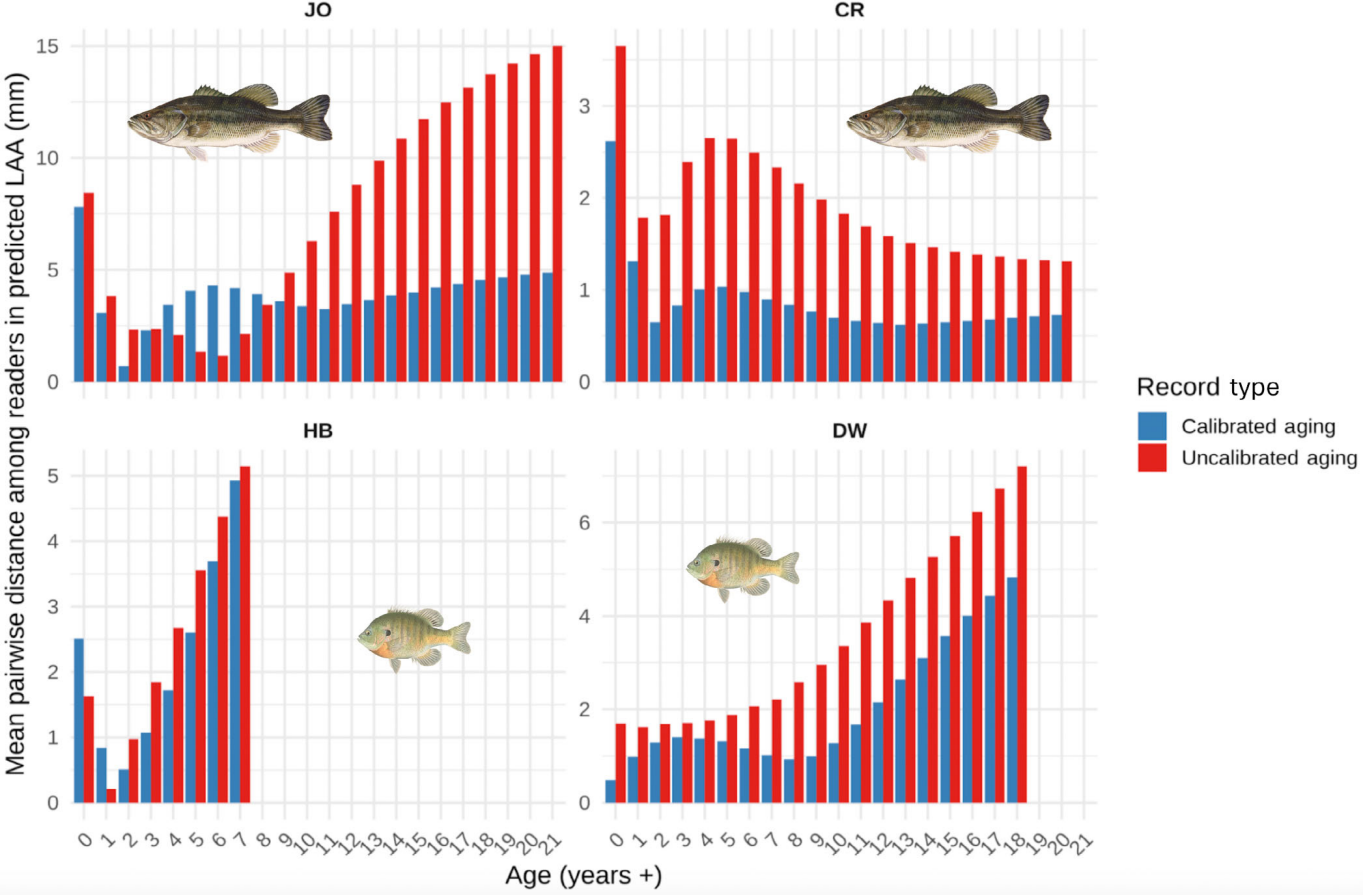
Mean pairwise distances among readers in predicted length‐at‐age (LAA) at all ages available for comparison, in each lake. JO, Jones Lake, largemouth bass; CR, Crampton Lake, largemouth bass; HB, Hummingbird Bog, bluegill; DW, Deadwood Lake, bluegill. In addition to reducing mean predicted LAA (Table 3), calibration appeared to substantially enhance the general convergence of readers on LAA metrics, suggesting that calibration may be additionally useful for reducing reader‐based variance effects in growth modelling. Age‐specific patterns in divergence differed among lakes, with some exhibiting greater reductions at older ages and others at younger ages, reflecting differences in how calibration‐induced shifts in age assignments influence the estimation of von Bertalanffy growth parameters and associated trajectories. Regardless of these differences in age‐specific structure, calibration consistently reduced overall pairwise divergence among readers across all lakes. Largemouth bass and bluegill illustrations used as figure labels were adapted from solrman (CC0 1.0), via Wikimedia Commons, and from the Freshwater and Marine Image Bank, University of Washington (public domain), respectively.

Furthermore, calibration resulted in substantial improvements in the convergence of readers on LAA metrics. Pairwise distances among individual readers' predicted LAA were significantly lower following calibration in three of four lakes. Reductions were strongest in Crampton Lake (*V* = 231, *n*[ages] = 21, *p* < 0.0001) and Deadwood Lake (*V* = 190, *n*[ages] = 19, *p* < 0.0001), and were also evident in Jones Lake (*V* = 217, *n*[ages] = 22, *p* = 0.0011). In Hummingbird Bog, calibration only marginally reduced divergence in predicted length‐at‐age among readers (*V* = 27, *n*[ages] = 8, *p* = 0.125), however, a substantially smaller number of ages was available for comparison (Figure 4).

## 4 DISCUSSION

While a multitude of studies have sought to quantify precision and accuracy differences among particular aging structures (Buckmeier & Howells, 2003; Isermann et al., 2010; Morehouse et al., 2013; Quist et al., 2007; Sotola et al., 2014), far fewer have directly interrogated the role of the reader in biases. To our knowledge, none have considered the structured cross‐referencing between different structures as a possible means of improving reader performance. Among centrarchid fishes, errors inherent to nonlethal aging structures (Long & Fisher, 2001) are typically assumed to propagate through demographic assessments as unavoidable and difficult to correct without switching to alternative structures (Isermann et al., 2010; Sotola et al., 2014). Dorsal spine‐based estimates, despite being generally less accurate than otolith‐derived estimates, have been reported in several instances to exhibit relatively high precision (reader agreement) compared to other nonlethal structures (Isermann et al., 2010; Morehouse et al., 2013; Sotola et al., 2014), and therefore provided a useful candidate structure for testing this assumption in age estimation. Ultimately, our results suggest that these errors are not immutable and instead can be meaningfully reduced through even limited, lake‐specific cross‐referencing of structures.

Importantly, our design, using paired otolith‐spine images, required readers to reconcile a fixed annulus count—derived from the otolith—with a spine in which some annuli may not be clearly expressed. This does not establish a direct visual correspondence between structures, but instead constrains how ambiguous or incomplete spine features are interpreted. As a result, calibration operates not only by providing a more ‘correct’ age, but by forcing resolution of uncertainty under degraded annulus signals, thereby reducing interpretive variability among readers.

In addition to shifting readers towards older age assignments, especially for older fish (Figure 3), calibration using lake‐specific paired otolith‐spine images reduced variability among readers in both individual spine age estimates and predicted growth trajectories. Thus, we found evidence not only of the expected reductions in traditional underestimation biases, but also of improvements in other important metrics of estimation quality. Importantly, although improvements in per‐estimate variability were ambiguous in Jones Lake, calibration still produced a marked convergence in predicted LAA metrics. Similarly, Hummingbird Bog showed only weak evidence of improved convergence in predicted LAA, but this likely reflects the small number of ages available for comparison, as we observed per‐estimate variability to improve strongly. The persistence of these effects across multiple rounds within the same lakes indicates that the improvements are related to lake‐specific biological patterns in annuli formation, rather than procedural learning or order effects (e.g. having appeared only once or decreasing in strength through rounds). Therefore, we suggest that the processes by which dorsal spines have been thought to yield underestimates, including early annuli resorption and vascular‐core obscuration (Erickson, 1983; Klein et al., 2017), may operate in at least partially lake‐specific ways and are potentially able to be learned by readers even with limited exposure to otoliths for cross‐referencing.

In general, our reader agreement metrics appear to fall near the range of values reported by other centrarchid studies. Among largemouth bass spines, Sotola et al. (2014) reported 30.8% complete agreement and 66.1% partial agreement (defined also as cases in which all reader ages existed within ±1 year) among four readers, slightly lower than both our uncalibrated (43.6% and 89.0%) and calibrated Figures (51.0% and 92.1%) among five readers. Other studies of largemouth bass reported complete and partial agreement values both lower (27.0% and 44.3% among three readers) (Morehouse et al., 2013) and higher (73.6% and 94.3% among two readers) (Lindelien et al., 2024) than our own. Klein et al. (2017) found the highest complete agreement (81% among three readers) among any of these studies. In other species, such as common carp (*Cyprinus carpio*, Linnaeus, 1758), complete agreement as high as 90.6% (among two readers) (Jackson et al., 2007) has been reported. We are not aware of any studies investigating the use of dorsal spines among bluegill.

Beyond the objective improvements observed in reader agreement and modelled growth trajectories, our results also provide insight into how readers perceived their own performance. Notably, calibration did not uniformly increase self‐reported confidence, despite producing gains in the quality of age estimates. Only two readers exhibited significant shifts in confidence, and these shifts were directionally inconsistent. Moreover, although confidence was weakly negatively correlated with CV, the effect was small to modest, indicating that readers' subjective certainty only provided limited information about their actual precision. Taken together, these results suggest that the benefits of calibration were not mediated by changes in perceived expertise or decisiveness, but instead that readers improved even when unaware or unconvinced that their performance had changed.

Although our results indicate that lake‐specific otolith calibration enhances the performance of spine‐derived estimates, calibrated spine readings are nevertheless expected to remain inferior to otolith readings in several important ways. First, calibration cannot recover information that has been fundamentally lost from the spine. Therefore, while the approach functions insofar as readers can be familiarized with faint annuli, and partially obscured remnants, it cannot restore early increments that have been completely erased (such that partial annuli components do not exist at all) and cannot therefore fully eliminate the structural limitations that underlie systematic underestimation in dorsal spines. Second, even where calibration appears to reduce underestimation biases, we found spine‐derived estimates to remain less precise than otoliths. Among otolith readings, mean CV was 1.3, well below the average among calibrated spine estimates (7.8), and our observed complete agreement rate of 87.9% similarly far exceeded the average rate among calibrated spine estimates (61.1%). Partial agreement was furthermore achieved on every otolith reading. Therefore, we emphasize that several fundamental advantages of otoliths remain, even compared to calibrated dorsal spine estimates.

Several limitations of our study warrant consideration. First, the use of only moderately experienced readers could be argued to amplify observed calibration effects through practice effects. However, the persistence of improvements across multiple rounds within the same lakes suggests that improvements are indeed related to lake‐specific biological patterns in annulus formation, and not to procedural learning. Moreover, as noted by Sotola et al. (2014), management agencies often rely on inexperienced or seasonal employees in high‐turnover roles for age estimation, suggesting that our findings are relevant in real managerial contexts. Second, the use of repeated measures may have induced image‐specific learning effects. However, psychological studies argue that this variety of short‐term, visual working memory is generally limited to 4–9 sequential images (Fukuda et al., 2010; Miller 1956), well below the sample size range (58–83 per lake, and in re‐randomized orders) employed in our study. Nonetheless, the possibility that small numbers of specific samples were recognized cannot be eliminated. Third, the small number of populations included could limit generalizability across natural systems. Yet, our study included two species, and size ranges from young‐of‐year to near‐asymptotic‐sized individuals, across both slow and fast‐growing populations, suggesting that calibration effects are likely to scale among centrarchid assessments. Other works have reported bluegill age ranges of 0–11 (Maryland Department of Natural Resources, n.d.; Niranjan Siriwardena, 2012) and largemouth bass age ranges of 0–23 (US Fish and Wildlife Service, n.d.). However, slightly older ages may be possible at northern latitudes, as in our study, where fish typically tend to have longer lifespans and exhibit slower growth (Braaten & Guy, 2002). Finally, while otoliths are considered the most accurate structure for centrarchid age determination, they independently exhibit some imperfections in accuracy and can exhibit small underestimations at older ages (e.g. 16+ years in largemouth bass, per Buckmeier & Howells, 2003), and at least one study suggests that the degree of underestimation may be inversely related to growth rates (Hoyer et al., 1985). Thus, our calibration is benchmarked against a high‐quality but nonetheless imperfect reference and provides definitive improvements only to the extent that otoliths represent true ages, which cannot be fully verified without comprehensive, within‐lake validation.

Here we demonstrate that small training sets, derived from paired, within‐lake otolith‐spine images, can serve as an effective and practical calibration tool for improving spine‐derived age estimates in centrarchid fishes. Specifically, we found that these structured training sets are useful in both reducing traditional underestimation biases and reducing reader‐based variance effects both in per‐estimate and growth modelling contexts. Therefore, we suggest that they offer a practical means of leveraging available incidental mortalities into otherwise nonlethal aging programs. Accordingly, we suggest that, where available, readers first be familiarized with lake‐specific paired otolith‐spine images before independently estimating spine ages. Further research should explore the generality of our structured training approach across a wider range of fish taxa and aging structures, including scales, fin rays and other nonlethal tissues, to determine whether similar calibration benefits can be realized in systems with different growth patterns or structural characteristics. Additionally, studies should quantify how calibration effects relate to true ages, rather than relying solely on comparisons to otolith‐derived estimates, to establish the absolute accuracy limits of calibrated nonlethal methods. Finally, experimental work should also investigate how calibration outcomes are influenced by training set characteristics, such as sample size and the distribution of ages within the set. Expanded investigation of these dimensions can better define the limits, scalability and efficacy of structured training as a means of reducing underestimation bias and improving the precision of nonlethal age estimates.

## AUTHOR CONTRIBUTIONS

M.P.W. conceptualized this research, collected specimens, analysed data and prepared the main draft. S.E.J. and J.T.‐D. provided supervision, provided input in study design and assisted with draft preparation. B.S.A. and A.N. collected specimens and assisted with draft preparation.

## FUNDING INFORMATION

The authors have nothing to report.

## CONFLICT OF INTEREST STATEMENT

The authors declare no conflict of interest.

## Data Availability

The data that support the findings of this study are openly available in OSF at https://osf.io/4k6jv.
